# Elemental Content in *Pleurotus ostreatus* and *Cyclocybe cylindracea* Mushrooms: Correlations with Concentrations in Cultivation Substrates and Effects on the Production Process

**DOI:** 10.3390/molecules25092179

**Published:** 2020-05-07

**Authors:** Georgios Koutrotsios, Georgios Danezis, Constantinos Georgiou, Georgios I. Zervakis

**Affiliations:** 1 Laboratory of General and Agricultural Microbiology, Agricultural University of Athens, Iera Odos 75, 11855 Athens, Greece; georgioskoutrotsios@gmail.com; 2 Laboratory of Chemistry, Agricultural University of Athens, Iera Odos 75, 11855 Athens, Greece; gdanezis@aua.gr

**Keywords:** trace element, edible mushroom, cultivation substrate, *Cyclocybe cylindracea*, *Pleurotus ostreatus*, medicinal mushroom, ICP-MS, dietary intake, bioconcentration factor

## Abstract

Few data exist about the effect of substrates’ elemental content on the respective concentrations in cultivated mushrooms, on the degradation of lignocellulosics or on production parameters. Sixteen elements (14 metals and 2 metalloids) were measured by inductively coupled plasma mass spectrometry (ICP-MS) in *Pleurotus ostreatus* and *Cyclocybe cylindracea* mushrooms, and in their seven cultivation substrates composed of various plant-based residues. Results revealed a high variability in elemental concentration among substrates which generally led to significant differences in the respective mushroom contents. High bioconcentration factors (BCFs) were noted for Cd, Cu, Mg and Zn for both species in all substrates. BCF of each element was variously affected by substrates’ pH, crude composition, and *p* and K content. Significant positive correlations were demonstrated for Cu, Fe, Mn and Li concentrations vs. a decrease of cellulose and hemicellulose in *P. ostreatus* substrates, and vs. mushrooms’ biological efficiency. In the case of *C. cylindracea*, Be, Mg and Mn concentrations were positively correlated with the decrease of hemicellulose in substrates, while a significant positive correlation was also recorded vs. mushroom productivity. Finally, it was found that 15% to 35% of the daily dietary needs in Mg, Se and Zn could be covered by mushroom consumption.

## 1. Introduction

Mushroom cultivation is a solid-state fermentation process during which agro-industrial residues are bioconverted into edible biomass of high nutritional value [1]. Mushrooms are characterized by their high protein and low calorie content, while they constitute a good source of vitamins, antioxidants and bioactive compounds, e.g., β-glucans, lectins, terpenoids, essential amino acids, statins and sterols, which demonstrate antitumor, antimicrobial, immunomodulatory, hypocholesterolemic, hypoglycemic, antithrombotic, anti-inflammatory, antiatherogenic, antihypertensive and prebiotic activities [2,3,4,5,6,7].

The organoleptic properties of mushrooms, combined with their benefits to human health, explain (partly) why they are among the agricultural products with the highest annual growth rate (7.5% per year [8]). Commercial cultivation of *Pleurotus ostreatus* (Jacq.) P. Kumm. for the production of ‘oyster’ mushrooms represents the fastest growing sector in this particular market [9], while cultivation of other species—such as *Cyclocybe cylindracea* (DC.) Vizzini and Angelini (autochthonous and widespread in Europe)—is expanding rapidly in Asia, Europe and the Americas. The most common substrates for these two species are widely accessible plant residues such as cereal straw and wood sawdust [1,10,11]. However, it has been shown that both could grow well and produce mushrooms on a large range of lignocellulosic residues and by-products; hence, they play an important role in managing/recycling organic wastes whose disposal is problematic, like those deriving from olive mills and wineries [12,13].

Fungi are heterotrophic organisms, and the nutritional value of mushrooms is inextricably related to the nature of their substrate. Recent studies have evidenced that their crude composition and their content in bioactive compounds vary significantly depending on the cultivation substrate used [12,14,15,16]. Trace elements, in particular, are essential for a plethora of fungal metabolic functions, including those related to enzyme production and degradation of lignocellulosics (e.g., Cu, Fe, Mn, Mo, Zn), while the biological role of several others is either inadequately resolved or associated (when in excess) with toxic effects on growth, reproduction and physiology [17,18,19,20,21]. Despite the existence of numerous publications reporting metal concentrations in wild mushrooms, considerably less information is available on the elemental composition of cultivated mushrooms. Most pertinent studies focused on their enrichment/biofortification with Cu, Fe, Li, Se and Zn [22,23,24,25,26,27,28], whereas only a few shed light on the effect of substrates in the elements’ content in the end-product [15,29,30]. In general, elements accumulation in mushrooms (wild or cultivated) was shown to be influenced by both exogenous and endogenous factors, including substrate [29,31,32], type of element [33,34,35], fungal lifestyle [36,37,38] and species [39,40,41].

In the present work, 16 elements (14 metals and two metalloids) were measured by inductively coupled plasma mass spectrometry (ICP-MS) in *P. ostreatus* and *C. cylindracea* mushrooms and in their cultivation substrates composed from various agricultural residues and agro-industrial by-products. Moreover, elemental concentrations in substrates were examined with respect to degradation of hemicellulose, cellulose and lignin as well as in relation to mushroom cultivation parameters. Finally, relationships between mushrooms’ crude composition and their content in various elements were investigated, the bioaccumulation factors were calculated, and dietary intake for each element was evaluated versus its respective needs/limitations.

## 2. Results and Discussion

### 2.1. Elemental Content in Cultivation Substrates

**Figure 1:**
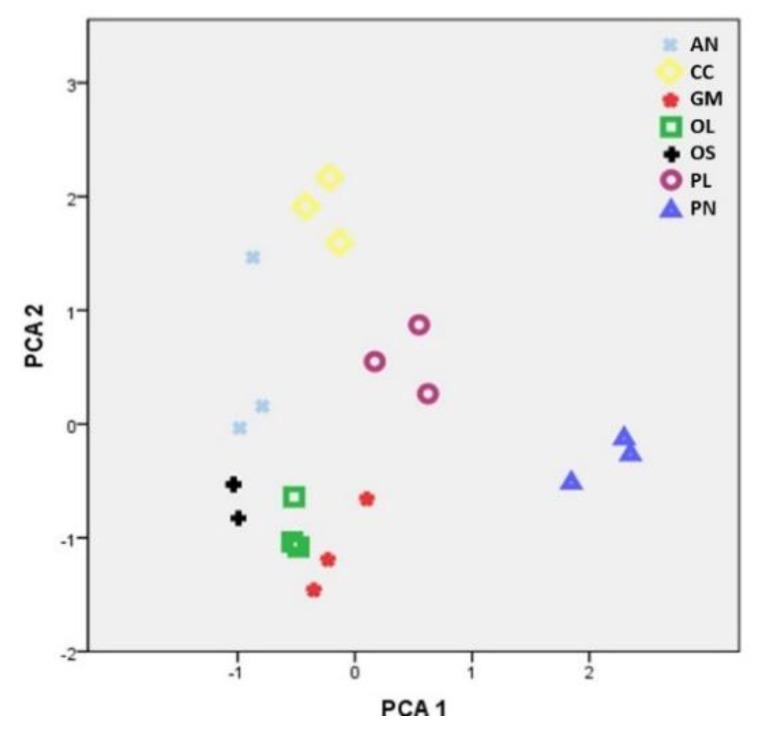
Discrimination of seven mushroom cultivation substrates through principal component analysis by using all element concentration data. Substrates used: AN: almond and walnut shells 1:1 *w*/*w*; CC: corn cobs; GM: grape marc plus cotton gin trash 1:1 *w*/*w*; OL: olive mill by-products (leaves and two-phase olive mill waste 1:1 *w*/*w*); OS: extracted olive-press cake; PL: date-palm tree leaves; PN: pine needles.

One of the critical factors affecting the concentration of elements in mushrooms is their availability in cultivation substrates. Concentrations for 16 elements are presented in seven mushroom substrates (Table 1). Among them, Ca presented relatively higher content in all samples examined (3.8–27.2 g·kg^−1^ d.w.), followed by Mg and Fe (0.3–3.9 g·kg^−1^ d.w. and 0.1–3 g·kg^−1^ d.w., respectively), whereas all other elements were detected in considerably lower amounts. In general, most of them presented a high variability in their content and statistically significant differences were observed in comparisons among substrates (Table 1). Of particular interest was that the pine needle substrate contained significantly higher concentrations of the majority of the elements examined, with the only exceptions Be, Mg, Mo, Ni and Se. This observation is in accordance with previous findings demonstrating strong bioaccumulation in the needles of pine species not only as regards heavy metals [42,43] but also with respect to rare earth elements [44]. Grape marc plus cotton gin trash was also rich in most of the elements studied, presenting the highest concentrations of Be and Mg, whereas extracted olive press cake, nut shells and olive mill by-products were the substrates with the lowest element concentrations (Table 1). According to the discriminant analysis performed, the latter three substrates are grouped together, whereas corn cobs, palm leaves and pine needles are characterized by high or moderate metal contents and form distinct groups (Figure 1). A wide range of elemental concentrations was also reported among substrates used for the cultivation of *C. cylindracea* and *Pleurotus eryngii* consisting of various wood sawdusts, supplemented or not with other lignocellulosic residues (e.g., corn cob, cottonseed hull) and cereal brans [30,32].

When the relationship between elements’ concentration (determined in this study) and crude composition (including P, K and Na, data deriving from Koutrotsios et al. [15]) of substrates was examined, significant correlations were evidenced for Mg vs. protein content (Pearson’s *r* = 0.89, *p* < 0.01) as well as for Se vs. P (*r* = 0.83, *p* < 0.05). Such results are in accordance with previous reports stating that most part of Mg accumulating in plant tissues is associated with protein synthesis by bridging ribosome subunits [45], while Se uptake is largely dependent on P availability [46].

### 2.2. Concentration of Elements in Mushrooms and Pertinent Correlations

The most abundant elements recorded in *C. cylindracea* and *P. ostreatus* mushrooms were Mg (1.29–2.80 g·kg^−1^ d.w.), followed by Ca (0.17–1.57 g·kg^−1^ d.w.). In addition, Fe and Zn were measured at high concentrations in both species (0.04–0.13 g·kg^−1^ d.w. and 0.05–0.12 g·kg^−1^ d.w., respectively; Table 2). No particular species-specific pattern was detected in respect to element accumulation; exceptions were Be (detected in consistently higher amounts in *C. cylindracea* than in *P. ostreatus*), and Fe, Mn, Se and Zn, which were measured in higher concentrations in *P. ostreatus* regardless of the substrate used (Table 2).

**Figure 2:**
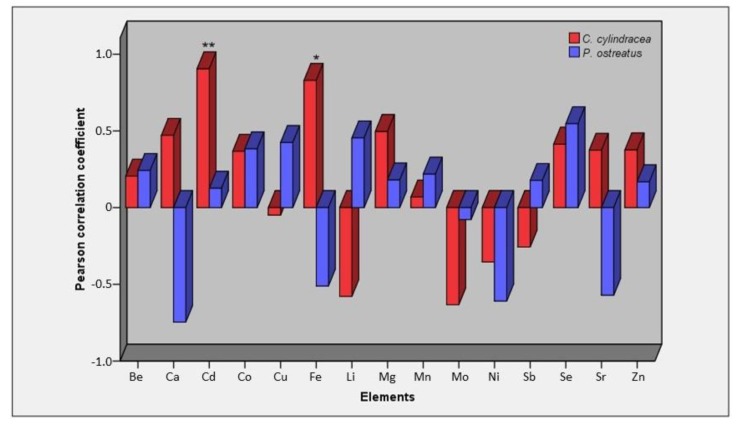
Values of Pearson’s *r* correlation coefficient for elements content in *C. cylindracea* and *P. ostreatus* mushrooms vs. their concentration in cultivation substrates. Levels of statistical significance are depicted as follows: *p* < 0.05 (*) and *p* < 0.01 (**).

Significant differences were recorded when elements’ concentrations in mushrooms from different substrates were compared, with the only exceptions of Li, Mo and Sb in *C. cylindracea* (Table 2). Such differences were further supported by the correlations detected between concentrations in substrates and in mushrooms; hence, each species presented a different accumulation pattern (Figure 2). Although both species exhibited a positive correlation for Be, Cd, Co, Mg, Se and Zn (*r* = 0.13–0.90) and negative for Mo and Ni (*r* = −0.07–0.63), opposite correlations were evidenced for Ca, Fe, Li, Sb and Sr when they were compared to each other. It is noteworthy that significant correlations were demonstrated only for Cd and Fe in *C. cylindracea* substrates and mushrooms (*r* = 0.90, *p* < 0.01 and *r* = 0.83, *p* < 0.05, respectively). As regards *P. ostreatus*, the highest correlation values were for Se (*r* = 0.55) and Li (*r* = 0.45). Both minerals are of particular importance to human health. Li is prescribed as a medication for bipolar disorder and treatment-resistant depression [47,48]; however, it exhibits toxic effects even at low concentrations, while its use is associated with nephrogenic diabetes insipidus [49,50]. On the other hand, Se forms part of selenoproteins, which have a wide range of pleiotropic effects, e.g., antioxidant/anti-inflammatory activities and enhancement of thyroid hormone production [51]. Therefore, Se enrichment of *P. ostreatus* cultivation substrates has recently attracted considerable research interest, especially towards determining its effect on mushrooms’ biological efficiency (i.e., mushrooms’ fresh weight over the substrates dry weight), antioxidant/antiviral properties and content of bioactive compounds [25,30,52].

In addition, a high variation in Ca concentration values was measured in mushrooms of both species examined (0.17–1.57 g·kg^−1^ d.w. in *P. ostreatus*, and 0.31–1.16 g·kg^−1^ d.w. in *C. cylindracea*); however, no correlation was detected vs. concentrations in the respective substrates. Since past studies reported that Ca content in mushrooms depends on the substrate composition [53,54], further experimental evidence is needed to clarify the mechanism of Ca absorption as well as possible interactions with other elements. It should be noted that the ionic charge density (i.e., electric charge of ions/ion size) was not found to correlate in any way with parameters examined in our study. Furthermore, *P. ostreatus* mushrooms produced on pine needles did not show significantly higher element concentrations when compared to most of the other substrates examined (with the only exceptions of As, Li and Se) although this particular medium was found to be considerably richer in elemental content, as previously stated. On the other hand, significantly higher amounts of Cd, Fe and Se were detected in *C. cylindracea* mushrooms derived from the pine needle substrate.

Element concentration values measured in *P. ostreatus* and *C. cylindracea* mushrooms in the frame of this study are within the range reported in the literature for both wild and cultivated mushrooms of these particular species [30,55,56,57,58,59,60,61,62]. A marked exception is the significantly higher concentration of Li in *P. ostreatus* (0.29–0.97 mg·kg^−1^) compared to values previously reported (0.04–0.21 mg·kg^−1^), which could be attributed to the higher Li content in the substrates examined in the present work (*r* = 0.45 for mushrooms vs. the corresponding substrates).

When pairwise comparisons were made among the elements measured in mushrooms of a given species, interesting correlations were revealed. Hence, both mushrooms exhibited significant *r* values between Sr and Ca (*r* = 0.97–0.98, *p* < 0.01), which can be explained by their similar chemical properties; such a correlation was also demonstrated when cultures of *Aspergillus terreus* were studied [63]. Especially as regards *P. ostreatus*, noteworthy correlations were established between Mo and Co (*r* = 0.90, *p* < 0.01) and between Ni and Fe (*r* = 0.94, *p* < 0.01). The latter was also reported for several wild edible species, i.e., *Agaricus bisporus*, *A. bitorquis*, *A. gennadii*, *Coprinus comatus*, *Psathyrella candolleana*, and *Volvopluteus gloiocephalus* [40]. Apparently, this particular interaction is quite common among basidiomycetes as it is in other organisms, e.g., plants, where it is involved in Ni toxicity and/or Fe deficiency [64,65,66]. In the case of *C. cylindracea*, high correlation values were recorded between Mo and Li (*r* = 0.97, *p* < 0.01) and Se and Zn (*r* = 0.92, *p* < 0.01), as well as between Fe and Cd (*r* = 0.96, *p* < 0.01); the latter is in agreement with previous reports on the high affinity of Fe oxides in the selective absorption of Cd [67,68]. In addition, positive correlations—albeit not statistically significant—were also recorded for Mn vs. Ni, Mn vs. Cu, Mn vs. Zn and Cu vs. Fe (*r* = 0.44–0.85) for both species examined, and are in accordance with the outcomes of relevant studies on wild edible mushrooms [40,69].

Although a large amount of literature information is available on metal content in mushrooms, the relative absence of data pertaining to interactions between them does not allow to infer broader pertinent conclusions. Noteworthy is also the correlation obtained between crude protein content (data deriving from Koutrotsios et al. [15]) and Cd concentration in *P. ostreatus* mushrooms (*r* = 0.88, *p* < 0.01). On the other hand, the findings of Chiu et al. [70] revealed that the addition of CdCl_2_ to wheat straw or to paper residues modified the amino acid profile and decreased their total content in *Pleurotus pulmonarius* mushrooms. Moreover, the significant correlation observed between crude protein content and Zn concentration in *C. cylindracea* mushrooms (*r* = 0.94, *p* < 0.01) is reported for the first time, and additional experimental data are required to interpret this relationship.

### 2.3. Bioconcentration Factors (BCFs)

**Figure 3:**
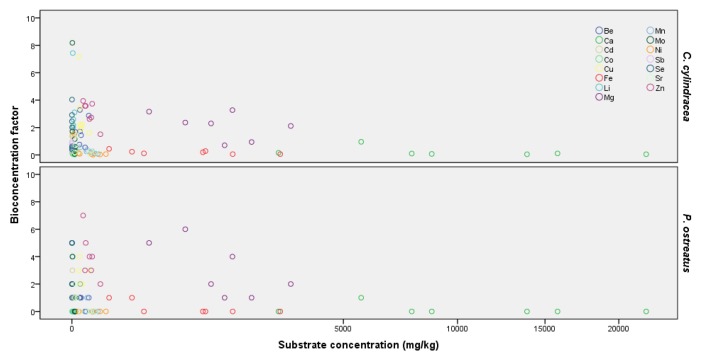
Scatter plot illustrating bioconcentration factors (mean values) of 15 elements in *Cyclocybe cylindracea* and *Pleurotus ostreatus* mushrooms produced on various substrates. AN: almond and walnut shells 1:1 *w*/*w*; CC: corn cobs; GM: grape marc plus cotton gin trash 1:1 *w*/*w*; OL: olive mill by-products (leaves and two-phase olive mill waste 1:1 *w*/*w*); OS: extracted olive-press cake; PL: date-palm tree leaves; PN: pine needles versus elemental concentration of substrates.

The BCFs for macro and trace elements were calculated as the ratio of their concentration in mushrooms to the respective content in the cultivation substrate. Living organisms demonstrating BCF values higher than 1.00 are defined as bioaccumulators and are considered efficient absorbers of elements. In the present study, BCF values ranged from 0.01 to 15.32 in *C. cylindracea* and from 0.01 to 9.87 in *P. ostreatus* (Figure 3).

**Figure 4:**
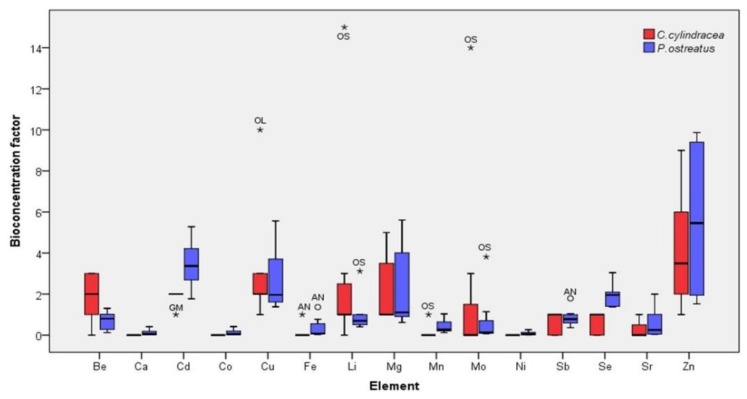
Box plot depicting bioconcentration factors of 15 elements in *Cyclocybe cylindracea* and *Pleurotus ostreatus* mushrooms produced on various substrates. AN: almond and walnut shells 1:1 *w*/*w*; CC: corn cobs; GM: grape marc plus cotton gin trash 1:1 *w*/*w*; OL: olive mill by-products (leaves and two-phase olive mill waste 1:1 *w*/*w*); OS: extracted olive-press cake; PL: date-palm tree leaves; PN: pine needles. The size of each box represents 50% of the values, the black horizontal line represents the median, and the error bars the greatest and least values. Circles and asterisks indicate extreme values higher than 1.5 and up to 3 times, and more than 3 times, respectively.

Average BCF values higher than 2.00 were noted for Cd, Cu, Mg and Zn in both mushroom species for all substrates, while the same values were calculated for Li in *C. cylindracea* and for Se in *P. ostreatus*. In contrast, average values up to 0.50 were recorded for Ca, Co and Ni for both species examined (Figure 4). In general, both species showed low BCFs when cultivated on substrates with high metal content and vice versa. Hence, significantly higher values were recorded for mushrooms produced on almond and walnut shells, on olive mill by-products and on extracted olive press cake, i.e., those with the lowest concentrations of most of the elements examined (Table 1; Figure 4). According to literature, BCFs for As and Cd tend to decrease as their respective substrate concentrations increase, indicating the possible presence of a regulatory mechanism [71,72]. In the present work, this was confirmed for Cd since mushrooms cultivated on substrates with high Cd content (i.e., pine needles followed by grape marc and corn cobs; Table 2) exhibited significantly lower BCF values than mushrooms cultivated on the other substrates.

In all cases, with the only exception of Cd in *C. cylindracea*, a negative correlation was established between BCFs and elements’ concentrations in the substrates, i.e., *r* = −0.38–0.85 and *r* = −0.17–0.90 for *P. ostreatus* and *C. cylindracea*, respectively. In addition, the BCF seems to be variously affected by the substrates’ pH, crude composition and macro element contents (the latter data on substrates’ properties derived from Koutrotsios et al. [15]). Thus, for both species, a significant positive correlation was observed between pH and BCFs for Cd (*r* = 0.60–0.90, *p* < 0.05) and Mg (*r* = 0.73–0.78, *p* < 0.05), which could be attributed to the presence of various metal binding ligands (e.g., humic and fulvic acids, polyphenols, chlorophyll), which keep these elements in solution despite their anticipated immobilization at high pH values. In contrast, a negative correlation was detected between P and BCFs for each one of the elements examined in this work, with the exception of Se (although in this case, most of the correlations were not significant). Furthermore, crude fat content was correlated with BCFs of Co (*r* = 0.77–0.83, *p* < 0.05), Li (*r* = 0.79–0.88, *p* < 0.05), Mn (*r* = 0.75–0.83, *p* < 0.05), Mo (*r* = 0.89–0.90, *p* < 0.01), Ni (*r* = 0.71–0.95, *p* < 0.05) and Zn (*r* = 0.91–0.92, *p* < 0.01), possibly as a result of their increased bioavailability. Finally, significant correlations were also obtained between BCF for Be and protein content in substrates of both species (*r* = 0.73–0.82, *p* < 0.05), and between BCF for Cd and K content in *C. cylindracea* substrates (*r* = −0.89, *p* < 0.01).

As concerns the impact of mushrooms’ composition, significant (negative) correlations were noted regarding P content vs. BCFs for Li, Mn, Mo and Zn in *C. cylindracea* (*r* = −0.63–0.79, *p* < 0.01), and vs. BCFs for Cu, Fe, Mn, Ni and Sb in *P. ostreatus* (*r* = −0.83–0.96, *p* < 0.01). This finding is indicative of the antagonistic relationship between P and the aforementioned elements as was previously reported in plants [65,73], while it also suggests that high P content might mitigate potential adverse effects related to the absorption of heavy metals by the fungus. The same type of correlation was recently reported between P and rare earth elements for the same mushroom species [44]. Moreover, the relationship existing between crude protein content and Se—through the pivotal role of the latter in the formation of selenoproteins (e.g., selenocysteine [74])—is confirmed by the findings of the present work, where a significant correlation was detected for both *C. cylindracea* and *P. ostreatus* (*r* = 0.70 and *r* = 0.78, *p* < 0.05, respectively).

### 2.4. Effect of Substrates Elements on Cultivation Parameters and Lignocellulosics Content

Correlation coefficients were also calculated to investigate the effect of individual elements on cultivation parameters and on mushrooms’ crude composition. In *P. ostreatus*, significant correlations were demonstrated for elements’ concentrations in substrates vs. hemicellulose (*r* = 0.63–0.87, *p* < 0.05; all elements) and cellulose (*r* = 0.53–0.87, *p* < 0.05; all elements except Mo and Ni) decrease, and consequently vs. mushroom yield expressed as biological efficiency (*r* = 0.47–0.91, *p* < 0.05). In the case of *C. cylindracea*, the sum of individual elements’ concentrations was found to have a positive effect on hemicellulose (*r* = 0.77, *p* < 0.05) and a negative effect on cellulose degradation (*r* = −0.58, *p* < 0.05). Significant positive correlations were observed between hemicellulose decrease and Be, Mg and Mn (*r* = 0.75–0.84, *p* < 0.05) concentrations, whereas negative correlations were noted between cellulose decrease and Ca), Cd and Sr (*r* = −0.70–0.76, *p* < 0.05) content in substrates. In addition, elements exhibiting a positive correlation to hemicellulose decrease (i.e., Be, Mg and Mn) also showed a significant positive correlation (*r* = 0.70–0.90, *p* < 0.05) with mushroom productivity (defined as the ratio of biological efficiency over the time length of the cultivation period [15]). The same trend was observed with Fe (*r* = 0.75, *p* < 0.05), Li (*r* = 0.90, *p* < 0.01) and Sr (*r* = 0.72, *p* < 0.05) concentrations. Finally, degradation of lignin was found to be (negatively) correlated with Ni content (*r* = −0.61 and *r* = −0.70 for *C. cylindracea* and *P. ostreatus*, respectively). These observations confirm that the presence of heavy metals (including Cd, Cr, Ni, Pb) confers reduced expression and/or inhibition of ligninolytic enzymes [19]. The functional role of metals in white-rot fungi, including *P. ostreatus,* has been extensively studied in the past, demonstrating the importance of some of them in mycelium growth and in the production of lignocellulolytic enzymes; such activities were found to be considerably affected by the presence of Cu, Mg, Mn and Ζn [75,76,77]. In the present work, concentrations of Cu, Fe, Mn and Li were found to be significantly higher in pine needles and grape marc as compared to the other substrates tested; this outcome could justify (at least partly) the greater decrease in hemicellulose and cellulose contents previously reported in the same cultivation media by the use of the same mushroom strains [15].

### 2.5. Consumption of Mushrooms—Dietary Intake

Metals classified as essential are involved in physiological processes, and their long-term deficiency leads to human diseases; among the rest, there are those which in low abundance/availability are not necessary for life and those for which only adverse/toxic effects are known so far [78]. Under this perspective, the estimated dietary intake (EDI) of each element as well as the percentage of the recommended or allowed daily dose were calculated through the consumption of *C. cylindracea* and *P. ostreatus* mushrooms (Table 3).

The high variability found in mushrooms’ elemental content resulted in a rather wide range in their EDI values. It is noteworthy that Se and Mo intake was calculated to vary (in respect to the recommended daily intake, RDI), depending on the substrate used, from 3.4% to 15.4% and from 0.6% to 26.1% in *C. cylindracea*, and from 12.9% to 35.2% and 1.4% to 7.4% in *P. ostreatus*, respectively. In addition, both *P. ostreatus* and *C. cylindracea* could considerably contribute to covering the dietary needs for Mg and Zn (up to 32–35% and 18–20%, respectively).

On the other hand, among the potentially harmful metals, the consumption of mushrooms examined in this study could lead to a Cd intake reaching up to 27–31% of the upper tolerance daily intake (TDI), whereas the intake of As and Ni is considerably lower (up to 3–4% of the TDI). According to the findings of the present and previous studies, heavy metal concentrations in cultivated *C. cylindracea* and *P. ostreatus* mushrooms are found at levels which do not raise any health-related concerns [54,85,86,87].

## 3. Materials and Methods

### 3.1. Biological Material and Mushroom Cultivation Substrates

*Cyclocybe cylindracea* strain LGAM 445 and *Pleurotus ostreatus* strain LGAM IK1123 were isolated from the wild, routinely preserved on potato dextrose agar (PDA, Difco) and maintained in the Culture Collection of the Agricultural University of Athens (Laboratory of General and Agricultural Microbiology). Both strains were previously cultivated in seven substrates [15], i.e., (i) nut (almond plus walnut) shells (1:1, *w*/*w*) [AN], (ii) corn cobs [CC], (iii) grape marc plus cotton gin trash (1:1, *w*/*w*) [GM], (iv) olive mill by-products (leaves plus two-phase olive mill waste 1:1, *w*/*w*) [OL], (v) extracted olive press-cake [OS], (vi) date palm leaves [PL], and (vii) pine needles [PN]. Four samples from each substrate (prior to cultivation) and four mushroom samples (from the first production flush of each treatment) were pre-frozen at −20 °C overnight and then freeze-dried in a Telstar Cryodos (Telstar Industrial, S.L., Terrassa, Spain) apparatus. Freeze-dried samples were ground to a fine powder and stored in the dark at −20 °C prior to analysis.

### 3.2. Chemicals and Standard Solutions

The chemicals used for analysis were nitric acid (Suprapur, 65% *w*/*v*; Merck, Darmstadt, Germany) and an inductively coupled plasma mass spectrometry (ICP-MS) multi-element standard (Sigma Aldrich, Saint Louis, MO, USA). Reversed osmosis ultra-pure water of 18.2 MΩ cm^−1^ resistance obtained from a MilliQ plus system (Millipore, Saint Quentin Yvelines, France) was used throughout.

### 3.3. Samples Preparation

Initially, complete digestion of samples was performed with a microwave-assisted system (CEM, Mars X-Press, Matthews, NC, USA). Samples (0.25 g) were soaked in 5 mL of concentrated HNO_3_ and were then heated in a microwave-accelerated digestion system as follows: the power was ramped from 0 to 960 W for 5 min and held constant for 15 min; the temperature reached a maximum of 175 °C, and a cool-down cycle followed for 15 min. Losses of volatile element compounds did not occur as the tubes were sealed during heating. The samples were then filtered with disposable syringe filters 0.20 μm/15 mm (Chromafil, Macherey-Nagel, Duren, Germany) and diluted tenfold with reversed osmosis ultra-pure water before injection into the ICP–MS instrument.

### 3.4. ICP-MS Analysis

The concentrations of 14 trace elements, (i.e., Fe, Co, Ni, Cu, Zn, As, Cd, Se, Sb, Be, Li, Mn, Mo, Sr) and two macro elements (Ca and Mg) were determined by using a Perkin Elmer (SCIEX, Markham, Ontario, Canada) Elan 9000 Series ICP-MS. Calibrations were obtained from 0.1 μg·kg^−1^ to 100 μg·kg^−1^. Measured values were higher than LOQ’s (limits of quantification) for all elements. In order to assess the accuracy of the analyses, standard reference materials were used: RM 8414 (bovine muscle powder), RM 8415 (whole egg powder), BCR-668 (mussel tissue) and ERM-BB 186 (pig kidney) for trace and macro elements. RM 8414 and RM 8415 were obtained from the National Institute of Standards & Technology (NIST), USA, while ERM-BB186 and BCR-668 were obtained from the Institute for Reference Materials and Measurements (IRMM), Belgium. Thus, satisfactory recoveries were obtained, generally in the range of 70–120%. Samples were analyzed in three batches; each batch included the reference materials, which were subjected to the exact same analytical process and were analyzed in triplicate.

### 3.5. Health Risk Assessment of Mushroom Consumption

The estimated daily intake of elements through mushroom consumption was calculated by the following equation [88]:

$$\mathrm{EDI}=\frac{\mathrm{CM}\times \mathrm{CR}}{\mathrm{BW}}$$

EDI (mg·kg^−1^·d^−1^) represents the estimated daily intake of elements, CM represents the concentration of elements in mushrooms (based on dry weight), CR corresponds to one serving of mushrooms (equal to 300 g of fresh weight, which contains 30 g of dry matter [89], and BW represents the body weight of adults (60 kg, in agreement with the EU Scientific Committee for the Food Adult Weight parameter [33]). Values of TDI (tolerance daily intake) and RDI (reference daily intake) derived from in-vivo experiments and estimates based on statistical data [79,80,81,82,83,84].

### 3.6. Statistical Analysis

Four replicates for each treatment were used in mushroom cultivation experiments. Determination of metals was performed in quadruplicate. Results are presented as mean ± standard deviation (SD). Analysis of variance followed by Gabriel’s t-test at 5% level of probability was performed to check differences between means through the use of SPSS (version 22, IBM) software. Pearson’s correlation coefficient (r) was employed to assess relationships (at significance levels of 0.05 and 0.01) between data obtained. Principal component analysis (PCA) was carried out by the same software to acquire an overview of the relationships among the parameters mentioned above.

## 4. Conclusions

The outcome of this study evidenced that the concentration of all elements exhibited high variability among various lignocellulosic by-products used as substrates, this resulting—in most cases—in significant differences in their respective content in mushrooms. Relatively high bioaccumulation factor values (BCF > 2.00) were noted for Cd, Cu, Mg and Zn by both species in all substrates; this was also the case for Li in *C. cylindracea* and for Se in *P. ostreatus*. In general, mushrooms showed low BCFs when cultivated on substrates with high metal content and vice versa. In addition, the BCF of each element was variously affected by substrates’ pH, crude composition and macro element contents. As regards the effect of substrates’ elemental content on cultivation parameters and lignocellulosics bioconversion, significant correlations were demonstrated for elements’ concentration in *P. ostreatus* substrates vs. the decrease in hemicellulose and cellulose (and consequently vs. biological efficiency). In the case of *C. cylindracea*, elements exhibiting a positive correlation to hemicellulose decrease (i.e., Be, Mg and Mn) also showed a significant positive correlation with productivity. Finally, degradation of lignin in substrates of both species was found to be (negatively) correlated with Ni content. It is noteworthy that the recommended daily intake of Se, Mg and Zn could be covered to a considerable extent by consuming the mushrooms examined.

## Figures and Tables

**Table 1:** Elements’ concentrations in the cultivation substrates used for the production of *Cyclocybe cylindracea* and *Pleurotus ostreatus* mushrooms. AN: almond and walnut shells 1:1 *w*/*w*; CC: corn cobs; GM: grape marc plus cotton gin trash 1:1 *w*/*w*; OL: olive mill by-products (leaves and two phase olive mill waste 1:1 *w*/*w*); OS: extracted olive press cake; PL: date palm tree leaves; PN: pine needles. Values [in mg·kg^−1^, except for Ca, Fe and Mg (g·kg^−1^); the latter are marked by an asterisk (*)] are expressed as means (standard deviation of means), *n* = 4. Lack of superscript letters in common indicates significant differences (Gabriel’s *t*-test, *p* < 0.05) when means corresponding to the same element are compared between substrates (i.e., comparisons along the same line).

Element	Substrate						
	AN	CC	GM	OL	OS	PL	PN
As	nd	nd	nd	nd	nd	0.10 (0.03) ^b^	2.62 (0.28) ^a^
Be	18.63 (5.76) ^cd^	2.47 (0.20) ^e^	74.25 (4.71) ^a^	20.73 (2.17) ^c^	13.30 (0.60) ^d^	18.95 (1.43) ^cd^	40.29 (1.93) ^b^
Ca*	7.11 (0.82) ^e^	3.88 (0.73) ^f^	15.38 (1.41) ^c^	18.28 (1.46) ^b^	3.79 (0.05) ^f^	9.55 (1.56) ^d^	27.21 (1.21) ^a^
Cd	0.09 (0.00) ^d^	0.12 (0.00) ^c^	0.14 (0.01) ^b^	0.09 (0.00) ^d^	0.09 (0.01) ^d^	0.12 (0.01) ^c^	0.23 (0.01) ^a^
Co	0.08 (0.01) ^d^	1.52 (0.25) ^b^	0.63 (0.17) ^c^	0.30 (0.01) ^d^	0.12 (0.00) ^d^	0.80 (0.09) ^c^	2.15 (0.31) ^a^
Cu	7.02 (0.63) ^c^	8.08 (0.56) ^c^	12.63 (0.54) ^b^	5.67 (0.34) ^c^	7.42 (0.54) ^c^	10.50 (1.93) ^b^	27.28 (2.21) ^a^
Fe*	0.09 (0.01) ^c^	1.16 (0.16) ^b^	1.49 (0.55) ^b^	0.36 (0.02) ^c^	0.14 (0.01) ^c^	1.15 (0.27) ^b^	2.98 (0.33) ^a^
Li	0.61 (0.06) ^c^	0.64 (0.00) ^c^	1.49 (0.14) ^b^	0.56 (0.10) ^c^	0.25 (0.02) ^d^	0.74 (0.09) ^c^	1.78 (0.16) ^a^
Mg *	1.02 (0.38) ^d^	0.46 (0.04) ^e^	3.88 (0.54) ^a^	1.69 (0.15) ^c^	0.26 (0.03) ^e^	2.04 (0.25) ^bc^	2.38 (0.26) ^b^
Mn	13.87 (1.24) ^bc^	23.14 (0.19) ^bc^	37.40 (1.48) ^bc^	41.32 (1.84) ^b^	7.88 (0.54) ^c^	43.76 (8.66) ^b^	97.40 (31.63) ^a^
Mo	2.54 (1.24) ^a^	2.33 (0.43) ^a^	0.88 (0.00) ^bc^	0.14 (0.03) ^c^	0.13 (0.01) ^c^	1.86 (0.18) ^ab^	1.84 (0.32) ^ab^
Ni	2.59 (0.09) ^c^	93.95 (3.48) ^a^	12.29 (1.40) ^c^	8.09 (0.08) ^c^	3.46 (0.30) ^c^	36.39 (2.82) ^b^	50.46 (17.35) ^b^
Sb	0.19 (0.01) ^de^	0.34 (0.02) ^c^	0.26 (0.02) ^cd^	0.28 (0.02) ^cd^	0.17 (0.03) ^e^	0.52 (0.04) ^b^	0.79 (0.09) ^a^
Se	0.11 (0.03) ^b^	0.14 (0.06) ^ab^	0.15 (0.04) ^ab^	0.13 (0.03) ^ab^	0.10 (0.01) ^b^	0.22 (0.05) ^a^	0.21 (0.06) ^a^
Sr	4.85 (0.60) ^e^	2.65 (0.22) ^e^	25.42 (2.21) ^c^	28.31 (2.41) ^b^	3.75 (0.27) ^e^	9.03 (1.48) ^d^	47.20 (1.38) ^a^
Zn	11.19 (4.85) ^d^	29.70 (0.24) ^bc^	15.40 (2.80) ^cd^	11.99 (1.29) ^d^	1.76 (0.29) ^d^	37.64 (10.93) ^b^	75.03 (14.22) ^a^

nd: not detected.

**Table 2:** Elements’ concentrations in *Cyclocybe cylindracea* LGAM 496 [*C.cl*] and *Pleurotus ostreatus* LGAM 1123 [*P.os*] mushrooms produced in seven cultivation substrates. AN: almond and walnut shells 1:1 *w*/*w*; CC: corn cobs; GM: grape marc plus cotton gin trash 1:1 *w*/*w*; OL: olive mill by-products (leaves and two phase olive mill waste 1:1 *w*/*w*); OS: extracted olive-press cake; PL: date palm tree leaves; PN: pine needles. Values [in mg·kg^−1^, except for Ca, Fe and Mg (g·kg^−1^); the latter are marked by an asterisk (*)] are expressed as means (standard deviation of means), *n* = 4. Lack of superscript letters in common indicates significant differences (Gabriel’s t-test, *p* < 0.05) when means corresponding to the same element are compared between substrates (i.e., comparisons along the same line).

Element		Substrate						
	Species	AN	CC	GM	OL	OS	PL	PN
As	*P.os*	0.03 (0.00) ^c^	0.01 (0.00) ^d^	0.07 (0.00) ^b^	nd	nd	nd	0.19 (0.03) ^a^
	*C.cl*	nd	nd	nd	nd	nd	nd	nd
Be	*P.os*	6.45 (2.49) ^bc^	3.23 (1.25) ^c^	15.53 (2.46) ^abc^	16.65 (9.91) ^ab^	12.31 (1.28) ^abc^	20.85 (7.25) ^a^	4.97 (0.52) ^bc^
	*C.cl*	10.37 (5.29) ^d^	7.74 (0.03) ^d^	41.39 (9.93) ^bc^	53.20 (0.10) ^ab^	31.12 (0.46) ^c^	62.81 (13.64) ^a^	15.47 (4.16) ^d^
Ca*	*P.os*	0.73 (0.09) ^ab^	1.02 (0.31) ^a^	0.17 (0.13) ^b^	0.29 (0.13) ^b^	1.57 (0.52) ^a^	0.52 (0.18) ^ab^	0.36 (0.06) ^b^
	*C.cl*	1.16 (0.02) ^a^	0.52 (0.08) ^c^	0.67 (0.31) ^abc^	0.63 (0.09) ^bc^	0.36 (0.08) ^c^	0.31 (0.02) ^c^	1.07 (0.41) ^ab^
Cd	*P.os*	0.38 (0.06) ^b^	0.62 (0.21) ^a^	0.38 (0.01) ^b^	0.39 (0.13) ^b^	0.30 (0.02) ^b^	0.31 (0.08) ^b^	0.41 (0.01) ^b^
	*C.cl*	0.20 (0.03) ^bc^	0.28 (0.03) ^b^	0.18 (0.01) ^c^	0.21 (0.02) ^bc^	0.21 (0.11) ^bc^	0.20 (0.01) ^bc^	0.54 (0.03) ^a^
Co	*P.os*	0.03 (0.00) ^c^	0.04 (0.01) ^abc^	0.03 (0.01) ^bc^	0.02 (0.00) ^c^	0.05 (0.02) ^a^	0.02 (0.01) ^c^	0.05 (0.00) ^ab^
	*C.cl*	0.02 (0.00) ^b^	0.02 (0.00) ^b^	0.03 (0.00) ^a^	0.02 (0.00) ^b^	0.02 (0.01) ^b^	0.01 (0.00) ^b^	0.03 (0.01) ^a^
Cu	*P.os*	39.05 (11.39) ^a^	15.86 (0.11) ^c^	21.13 (1.94) ^bc^	18.97 (1.36) ^bc^	30.16 (6.90) ^ab^	16.33 (5.87) ^c^	37.72 (1.89) ^a^
	*C.cl*	23.88 (2.33) ^b^	18.83 (1.75) ^b^	24.08 (2.70) ^b^	57.55 (35.77) ^a^	25.47 (0.60) ^b^	19.02 (2.13) ^b^	32.83 (2.90) ^b^
Fe *	*P.os*	0.13 (0.01) ^a^	0.08 (0.01) ^bc^	0.09 (0.02) ^abc^	0.12 (0.02) ^ab^	0.11 (0.01) ^abc^	0.08 (0.02) ^c^	0.10 (0.01) ^abc^
	*C.cl*	0.05 (0.01) ^b^	0.07 (0.00) ^b^	0.04 (0.00) ^b^	0.05 (0.00) ^b^	0.04 (0.01) ^b^	0.05 (0.01) ^b^	0.10 (0.03) ^a^
Li	*P.os*	0.29 (0.04) ^b^	0.63 (0.06) ^ab^	0.61 (0.28) ^ab^	0.56 (0.21) ^ab^	0.79 (0.17) ^ab^	0.52 (0.04) ^ab^	0.97 (0.55) ^a^
	*C.cl*	0.95 (0.07) ^a^	0.39 (0.16) ^a^	1.15 (0.12) ^a^	1.63 (1.21) ^a^	3.87 (2.35) ^a^	1.05 (0.15) ^a^	0.37 (0.08) ^a^
Mg *	*P.os*	2.80 (0.04) ^a^	2.44 (0.65) ^ab^	2.40 (0.40) ^ab^	1.87 (0.12) ^bc^	1.47 (0.30) ^c^	1.94 (0.36) ^bc^	2.08 (0.11) ^abc^
	*C.cl*	2.56 (0.53) ^a^	1.85 (0.24) ^bc^	2.26 (0.31) ^ab^	1.71 (0.03) ^bc^	1.29 (0.14) ^c^	2.04 (0.29) ^ab^	2.21 (0.37) ^ab^
Mn	*P.os*	13.76 (0.85) ^a^	6.27 (2.00) ^d^	11.06 (1.33) ^abc^	10.33 (0.72) ^bc^	8.14 (1.46) ^cd^	7.05 (1.45) ^d^	11.70 (0.7) ^ab^
	*C.cl*	5.64 (0.57) ^ab^	4.71 (1.28) ^ab^	5.13 (1.03) ^ab^	6.36 (1.32) ^a^	5.20 (0.15) ^ab^	4.26 (0.65) ^b^	5.42 (1.07) ^ab^
Mo	*P.os*	0.19 (0.03) ^ab^	0.29 (0.02) ^ab^	0.09 (0.02) ^b^	0.16 (0.08) ^ab^	0.48 (0.11) ^a^	0.16 (0.03) ^ab^	0.49 (0.36) ^a^
	*C.cl*	0.42 (0.27) ^a^	0.07 (0.05) ^a^	0.31 (0.03) ^a^	0.42 (0.28) ^a^	1.74 (0.98) ^a^	0.04 (0.02) ^a^	0.06 (0.02) ^a^
Ni	*P.os*	0.69 (0.21) ^ab^	0.41 (0.02) ^ab^	0.52 (0.22) ^ab^	0.83 (0.43) ^a^	0.59 (0.15) ^ab^	0.28 (0.08) ^b^	0.49 (0.05) ^ab^
	*C.cl*	0.34 (0.12) ^c^	0.41 (0.09) ^bc^	0.55 (0.06) ^abc^	0.71 (0.06) ^a^	0.65 (0.31) ^ab^	0.49 (0.07) ^abc^	0.58 (0.17) ^abc^
Sb	*P.os*	0.35 (0.07) ^a^	0.32 (0.01) ^a^	0.21 (0.03) ^bc^	0.22 (0.04) ^bc^	0.17 (0.03) ^c^	0.22 (0.03) ^bc^	0.29 (0.10) ^ab^
	*C.cl*	0.18 (0.01) ^a^	0.17 (0.03) ^a^	0.23 (0.04) ^a^	0.26 (0.16) ^a^	0.20 (0.04) ^a^	0.21 (0.04) ^a^	0.18 (0.03) ^a^
Se	*P.os*	0.34 (0.05) ^ab^	0.29 (0.01) ^abc^	0.21 (0.11) ^bc^	0.27 (0.01) ^abc^	0.15 (0.09) ^c^	0.30 (0.08) ^abc^	0.41 (0.00) ^a^
	*C.cl*	0.17 (0.01) ^ab^	0.07 (0.01) ^c^	0.08 (0.02) ^c^	0.07 (0.00) ^c^	0.04 (0.03) ^c^	0.10 (0.02) ^bc^	0.18 (0.07) ^a^
Sr	*P.os*	2.57 (0.48) ^bc^	3.93 (1.04) ^b^	1.25 (0.65) ^c^	1.40 (0.34) ^c^	7.49 (2.75) ^a^	2.27 (0.68) ^bc^	1.97 (0.21) ^bc^
	*C.cl*	3.79 (0.08) ^a^	2.19 (0.24) ^abc^	2.12 (0.17) ^abc^	2.02 (0.98) ^bc^	1.43 (0.22) ^c^	1.33 (0.10) ^c^	3.59 (1.37) ^ab^
Zn	*P.os*	110.41 (4.07) ^a^	96.15 (24.74) ^ab^	118.26 (2.87) ^a^	112.70 (1.00) ^a^	73.87 (2.19) ^b^	73.38 (11.25) ^b^	114.15 (6.36) ^a^
	*C.cl*	104.29 (1.69 ^a^	50.48 (0.46) ^c^	76.65 (11.19) ^b^	74.63 (5.16) ^b^	54.18 (13.61) ^c^	72.51 (2.05) ^b^	99.46 (4.89) ^a^

nd: not detected.

**Table 3:** Elements’ estimated daily intake (EDI, mg·kg^−1^·d^−1^) and percent coverage of upper tolerable or recommended level of each element’s intake (in parentheses) through the consumption of one serving (calculated as 300 g f.w. corresponding to 30 g d.w.) of *C. cylindracea* and *P. ostreatus* mushrooms by an adult person (60 kg). TDI: tolerance daily intake (mg·day^−1^); RDI: Recommended dietary intake (mg·day^−1^).

Element	*C. cylindracea*	*P. ostreatus*	TDI	RDI	Reference
As	nd	nd	0.128		[79]
Be	0.23–1.88 (0.8–6.8%)	0.10–0.63 (0.4–2.3%)	27.6		[80]
Ca	9.21–34.90 (0.9–3.5%)	5.11–47.0 (0.5–4.7%)	nr	1000	[81]
Cd	0.01–0.02 (9.0–27.0%)	0.01–0.02 (15.0–31.0%)	0.060		[79]
Co	0.00–0.00	0.00–0.00	nr		
Cu	0.56–1.73 (5.7–17.3%)	0.48–1.17 (4.8–11.7%)	10	2.2	[79]
Fe	1.07–2.98 (3.6–9.9%)	2.40–3.76 (8.0–12.5%)	48	10–50	[79]
Li	0.01–0.12 (1.1–11.6%)	0.01–0.03 (0.9–2.9%)	nr	1	[82]
Mg	38.85–76.65 (16.2–31.9%)	44.07–84.14 (18.4–35.1%)	nr	240	[81]
Mn	0.13–0.19 (4.3–6.4%)	0.19–0.41 (6.3–13.8%)	11	3	[79,83]
Mo	0.00–0.05 (0.6–26.1)	0.00–0.01 (1.4–7.4%)	nr	0.1–0.3	[79]
Ni	0.01–0.02 (1.4–3.0%)	0.01–0.02 (1.2–3.5%)	0.720		[79]
Sb	0.01–0.01 (1.4–2.2%)	0.01–0.01 (1.4–2.9%)	0.36		[79]
Se	0.00–0.01 (3.4–15.4%)	0.00–0.01 (12.9–35.1)	320–480	0.026–0.035	[79]
Sr	0.04–0.11 (0.0–0.0%)	0.04–0.22 (0.0–0.0%)	2400		[84]
Zn	1.51–3.13 (8.7–17.9%)	2.20–3.55 (12.6–20.3%)	60	15–20	[79]

nd: not detected; nr: not referred.
